# Watchful waiting is an acceptable treatment option for asymptomatic primary ocular adnexal mucosa‐associated lymphoid tissue lymphoma: A retrospective study

**DOI:** 10.1002/cam4.5237

**Published:** 2022-09-12

**Authors:** Kentaro Mizuhara, Tsutomu Kobayashi, Mitsushige Nakao, Ryoichi Takahashi, Hiroto Kaneko, Kazuho Shimura, Koichi Hirakawa, Nobuhiko Uoshima, Katsuya Wada, Eri Kawata, Reiko Isa, Takahiro Fujino, Taku Tsukamoto, Shinsuke Mizutani, Yuji Shimura, Akiko Yoneda, Akihide Watanabe, Chie Sotozono, Junya Kuroda

**Affiliations:** ^1^ Division of Hematology and Oncology Kyoto Prefectural University of Medicine Kyoto Japan; ^2^ Department of Hematology Otsu City Hospital Otsu Shiga Japan; ^3^ Department of Hematology Omihachiman Community Medical Center Omihachiman Shiga Japan; ^4^ Department of Hematology Aiseikai Yamashina Hospital Kyoto Japan; ^5^ Department of Hematology Fukuchiyama City Hospital Kyoto Japan; ^6^ Department of Hematology Japanese Red Cross Kyoto Daini Hospital Kyoto Japan; ^7^ Department of Hematology Matsushita Memorial Hospital Osaka Japan; ^8^ Department of Ophthalmology Kyoto Prefectural University of Medicine Kyoto Japan

**Keywords:** asymptomatic patients, primary ocular adnexal marginal zone lymphoma of mucosa‐associated lymphoid tissue, radiotherapy, rituximab monotherapy, watchful and waiting

## Abstract

**Background:**

Primary ocular adnexal mucosa‐associated lymphoid tissue (MALT) lymphoma (POAML) is the most common subtype of indolent ocular adnexal lymphomas. Although radiotherapy (RT) is the standard of care for localized POAML, it can occasionally lead to permanent side effects. Other treatment strategies, such as rituximab (R) monotherapy and immunochemotherapy, have been used for POAML treatment, but their long‐term benefits and relative merits remain unclear. While watchful waiting (WW) is a potential option for some indolent lymphomas, the benefits of WW for POAML patients are also unclear.

**Methods:**

We here retrospectively analyzed 75 patients who were diagnosed with POAML between 2008 and 2019 in the institutions of the Kyoto Clinical Hematology Study Group.

**Results:**

Commonly involved sites were conjunctiva (42.7%), orbit (36.0%), and lacrimal gland (12.0%), and most patients (92.0%) presented with Ann Arbor stage IE disease. The treatment strategy was selected at the physicians' discretion. More patients without subjective symptoms by tumor mass were subjected to WW (29 patients), while more patients with tumor‐derived subjective symptoms were treated by tumor‐directed therapy (24 received focal RT, and 19 received R monotherapy). Complete response rates were 79.2% and 42.1% in the RT and R groups, respectively. At 60 months of follow‐up, the estimated proportions of POAML patients not requiring new treatment were 69.4%, 85.2%, and 53.8% in the WW, RT, and R groups, respectively. There were no significant differences in the time to start a new treatment between WW and RT groups (median: both not reached [NR], *p* = 0.187) and between WW and R groups (median: NR vs. 69.0 months, *p* = 0.554). No specific predictive factor for the future need of treatment was identified in the WW group.

**Conclusion:**

Our results demonstrate WW may be an acceptable treatment option for POAML, especially for asymptomatic patients.

## Graphical abstract

Watchful waiting is expected to produce no inferior time to next treatment (A) and progression free survival (B) compared to radiation or rituximab monotherapy in patients with primary ocular adnexal mucosa‐associated lymphoid tissue lymphoma, especially those without eye‐related symptom.

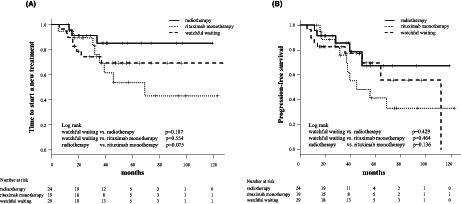

## 1 INTRODUCTION

Primary ocular adnexal marginal zone lymphoma of mucosa‐associated lymphoid tissue (MALT) (POAML) is the most frequent subtype of ocular adnexal lymphomas, accounting for 80%–90% of all ocular adnexal lymphomas in East Asia^1^, ^2^ and 60% in Western countries.^3^ POAML patients generally experience an indolent clinical course and achieve long‐term survival, with the 10‐year disease‐specific survival exceeding 90%.^4^, ^5^, ^6^, ^7^, ^8^

Radiotherapy (RT) is widely recognized as the standard of care for localized POAML because of its high efficacy for local control and tolerability.^9^, ^10^, ^11^, ^12^, ^13^, ^14^, ^15^, ^16^ However, complications, such as cataracts, retinitis, dry eye, and optic neuropathy, of RT occasionally lead to irreversible damage.^12^, ^13^, ^14^, ^15^, ^16^ Other treatment strategies, such as rituximab monotherapy,^17^, ^18^, ^19^, ^20^ immunochemotherapy,^21^ intralesional rituximab,^22^ and antibiotic therapy for *Chlamydia psittaci*,^23^ have been used for POAML treatment, but their long‐term benefits and the superiority of one strategy over others are unclear. Considering no significant difference in overall survival (OS) between watchful waiting (WW) and rituximab monotherapy or immunochemotherapy in asymptomatic patients with advanced‐stage, low‐grade lymphomas, such as follicular lymphoma,^24^, ^25^, ^26^ WW is expected to be a therapeutic option for POAML, too; however, the benefits of WW for POAML patients are unclear.

To answer this clinical question, we retrospectively investigated the disease characteristics and treatment outcomes of POAML patients diagnosed and treated in the institutes of the Kyoto Clinical Hematology Study Group (KOTOSG).

## 2 METHODS

### 2.1 Study design

We retrospectively analyzed the background, disease status, treatments, and clinical outcomes of patients histologically diagnosed with POAML between February 2008 and September 2019 at institutes belonging to the KOTOSG. The primary ocular adnexal disease sites were defined as involvement of the conjunctiva, orbit, eyelid, or lacrimal gland. The disease stage according to the Ann Arbor staging system was evaluated via ophthalmological and physical examination, computed tomography (CT), magnetic resonance imaging (MRI), positron emission tomography CT (PET‐CT), or bone marrow analysis. Bilateral ocular adnexal lymphoma without other lymphomatous involvement was defined as stage IE disease. Patients with a follow‐up period of <1 year were excluded from the analysis.

Clinical data of each patient were collected using the patient's case report form including background factors: age, gender, Eastern Cooperative Oncology Group performance status (PS), and relevant medical history, including symptoms, involved sites, International Prognostic Index (IPI), MALT‐IPI,^27^ serological laboratory data, imaging data, clinical stage, and details of treatment (the degree of surgical resection, types of initial treatment, response to treatment, survival periods, and adverse events [AEs]).

### 2.2 Analysis of treatment outcomes

The time to start a new treatment (TTNT), progression‐free survival (PFS), OS, overall response rate (ORR), complete response (CR) rate, and AEs of treatment were analyzed. The TTNT was defined as the interval from the commencement of treatment to the date of starting next‐line treatment, including systemic chemotherapy or RT. In the case of WW, the TTNT was defined as the time from diagnosis to the initiation of a new treatment. In patients without the need for a new treatment, survival data were censored at the date last assessed. The PFS was defined as the interval from the commencement of treatment to disease progression or death, and the OS was defined as the interval from the commencement of treatment to death. In the case of WW, the PFS and OS were calculated from the date of diagnosis. The PFS was censored at the start of a new treatment in case patients proceeded to new treatment without disease progression. The Lugano 2014 criteria were used for response assessment.^28^ AEs were graded using the National Cancer Institute Common Terminology Criteria for Adverse Events (CTCAE) version 5.0. The treatment response of ocular lesions and ocular AEs were assessed by at least two physicians, including a hematologist and an ophthalmologist.

### 2.3 Statistical analysis

The distribution of patient characteristics between groups was compared using Student's *t*‐test and the Kruskal–Wallis test for continuous variables and Fisher's exact test for qualitative variables. The Kaplan–Meier method was performed for survival analysis, with a log‐rank test for comparison of survival curves. The confidence interval (CI) was 95%, and *p* < 0.05 was considered statistically significant. All statistical analyses were performed using Easy R version 1.37.^29^

## 3 RESULTS

### 3.1 Patient characteristics and clinical presentation in association with the treatment procedure

Overall, 103 patients were diagnosed with POAML, of which 11 were excluded from analysis because of lost follow‐up, 11 due to missing data, and six due to short follow‐up within 1 year. As a result, 75 patients were analyzed in this study. Patient characteristics are shown in Table 1. The median age was 68 years (range: 26–92 years), and 41 patients (54.7%) were females. The most commonly involved sites were the conjunctiva (42.7%), followed by the orbit (36.0%), lacrimal gland (12.0%), and eyelid (8.0%). Of the 75 patients, 69 (92.0%) presented with stage IE disease, followed by 3 (4.0%) with stage IVE, 2 (2.7%) with stage IIE, and 1 with stage IIIE. In addition, 61 patients (81.3%) presented with a unilateral ocular region, 14 (18.7%) presented with bilateral disease regions, 5 (6.7%) had complications with an immunoglobulin G4 (IgG4)‐related disease, and four of them had lymphoma involvement in the lacrimal gland. According to the IPI, most patients (69, 92.0%) were classified as low risk, three patients (4.0%) as low‐intermediate risk, 2 (2.7%) as high‐intermediate risk, and 1 (1.3%) as high risk. According to MALT‐IPI, 36 (48.0%) of 75 patients were classified as low risk, 34 patients (45.3%) as low‐intermediate risk, and only 5 (6.6%) as high risk. The most frequent complaint at the initial presentation was the recognition of a tumor without symptoms in 29 patients, followed by swelling in 19, hyperemia in nine, discomfort in seven, and other symptoms in 11. At tumor biopsy for histopathologic diagnosis, the tumor was completely resected in 17 patients, while a residual tumor existed after biopsy in 58 patients (Table 1).

**TABLE 1:** Patient characteristics

	Total (*n* = 75)	WW (*n* = 29)	Radiotherapy (*n* = 24)	Rituximab monotherapy (*n* = 19)	Others (*n* = 3)	*p*
Median age (range)	68 (26–92)	63 (37–92)	68 (26–85)	68 (58–81)	67 (59–78)	0.044†
Gender, *n* (%)						0.373*
Male/Female	34 (45) / 41(55)	10 (34) / 19 (66)	14 (58) / 10 (42)	9 (47) / 10 (53)	1 (33) / 2 (67)	
Involved site, *n* (%)						<0.001*
Conjunctiva	32 (43)	19 (66)	5 (21)	8 (42)	0	
Orbit	27 (36)	3 (10)	13 (54)	9 (47)	2 (67)	
Lacrimal gland	9 (12)	5 (17)	2 (8)	2 (11)	0	
Eyelid	6 (8)	1 (3)	4 (17)	0	1 (33)	
Lacrimal caruncle	1 (1)	1 (3)	0	0	0	
Laterality, *n* (%)						0.076*
Unilateral/Bilateral	61 (81) / 14 (19)	24 (83) / 5 (17)	22 (92) / 2 (8)	14 (74) / 5 (26)	1 (33) / 2 (67)	
Degree of resection, *n* (%)						<0.001*
Complete resection	17 (23)	14 (48)	1 (4)	2 (11)	0	
Partial resection	58 (77)	15 (52)	23 (96)	17 (89)	3 (100)	
Medical history, *n* (%)						
IgG4‐related disease	5 (7)	3 (10)	1 (4)	1 (5)	0	
Rheumatoid Arthritis	1 (1)	0	0	0	1 (33)	
Sjogren's syndrome	2 (3)	0	0	0	2 (67)	
Ann Arbor Staging, *n* (%)						0.047*
IE	69 (92)	28 (97)	23 (96)	16 (84)	2 (67)	
IIE	2 (3)	1 (3)	0	1 (5)	0	
IIIE	1 (1)	0	0	0	1 (33)	
IVE	3 (4)	0	1 (4)	2 (11)	0	
IPI, *n* (%)						0.054*
Low	69 (92)	29 (100)	22 (92)	16 (84)	2 (67)	
Low‐intermediate	2 (3)	0	1 (4)	1 (5)	0	
High‐intermediate	3 (4)	0	1 (4)	2 (11)	0	
High	1 (1)	0	0	0	1 (33)	
MALT‐IPI, *n* (%)						
Low	36 (48)	18 (62)	12 (50)	6 (32)	0	0.040*
Intermediate	34 (45)	11 (68)	11 (46)	10 (52)	2 (67)	
High	5 (7)	0	1 (4)	3 (16)	1 (33)	
Presentation, *n* (%)						
Tumor recognition	29 (39)	13 (45)	9 (38)	6 (32)	1 (33)	
Swelling	19 (25)	7 (24)	8 (33)	3 (16)	1 (33)	
Discomfort	7 (9)	6 (21)	0	1 (5)	0	
Hyperemia	9 (12)	3 (10)	0	6 (32)	0	
Ptosis	6 (8)	0	4 (17)	1 (5)	1 (33)	
Diplopia	3 (4)	0	1 (4)	2 (11)	0	
Proptosis	1(1)	0	1 (4)	0	0	
Incidental	1 (1)	0	1 (4)	0	0	

Abbreviations: IPI, International Prognostic Index; POAML, primary ocular adnexal marginal zone lymphoma of mucosa‐associated tissue.

* Fisher's exact test.

^†^ Kruskal–Wallis test.

WW was the most frequent first‐line modality, selected for 29 (38.7%) of 75 patients, including 14 patients with complete tumor resection at biopsy. In the remaining 46 patients (61.3%), the primary treatment modalities were RT in 24 (52.0%) (RT group), rituximab monotherapy in 19 (41.3%) (R group), and other modalities in 3 (6.5%), including systemic chemotherapy with rituximab plus cyclophosphamide, doxorubicin, vincristine, and prednisolone (CHOP)‐like chemotherapy in two patients and discontinuation of methotrexate in one patient with rheumatoid arthritis.

Of the 29 patients in the WW group, 19 (65.5%) had primary lesions in the conjunctiva, which was more frequent compared with the RT or rituximab group. In addition, the tumor was completely resected in 14 patients (48.3%) at diagnosis, which was also more frequent compared with the RT or R group. All patients except one had stage IE disease in the WW group. Although 6 (20.7%) of the 29 patients complained of eye discomfort with a foreign‐body sensation caused by lymphoma, they showed no treatment‐emergent symptom that could lead to vision impairment, such as ptosis, diplopia, or proptosis.

In the RT and R groups, 43 of the 46 patients had a residual tumor after biopsy. Six (25.0%) of 24 patients in the RT group and 3 (15.8%) of 19 patients in the R group had treatment‐emergent subjective symptoms that potentially impair visual function. In these groups, the orbit was the most frequent site of lymphoma. The two‐wedge oblique technique was used for our patients with POAML analyzed in this study. The median doses of total and fractional radiation were 30 Gy (range: 24–30 Gy) and 2 Gy (range: 1.5–2 Gy), respectively. Rituximab was administered intravenously at a dose of 375 mg/m^2^ once weekly up to eight doses. Patients with bilateral disease or advanced disease tended to undergo rituximab monotherapy.

### 3.2 Treatment response

**FIGURE 1:**
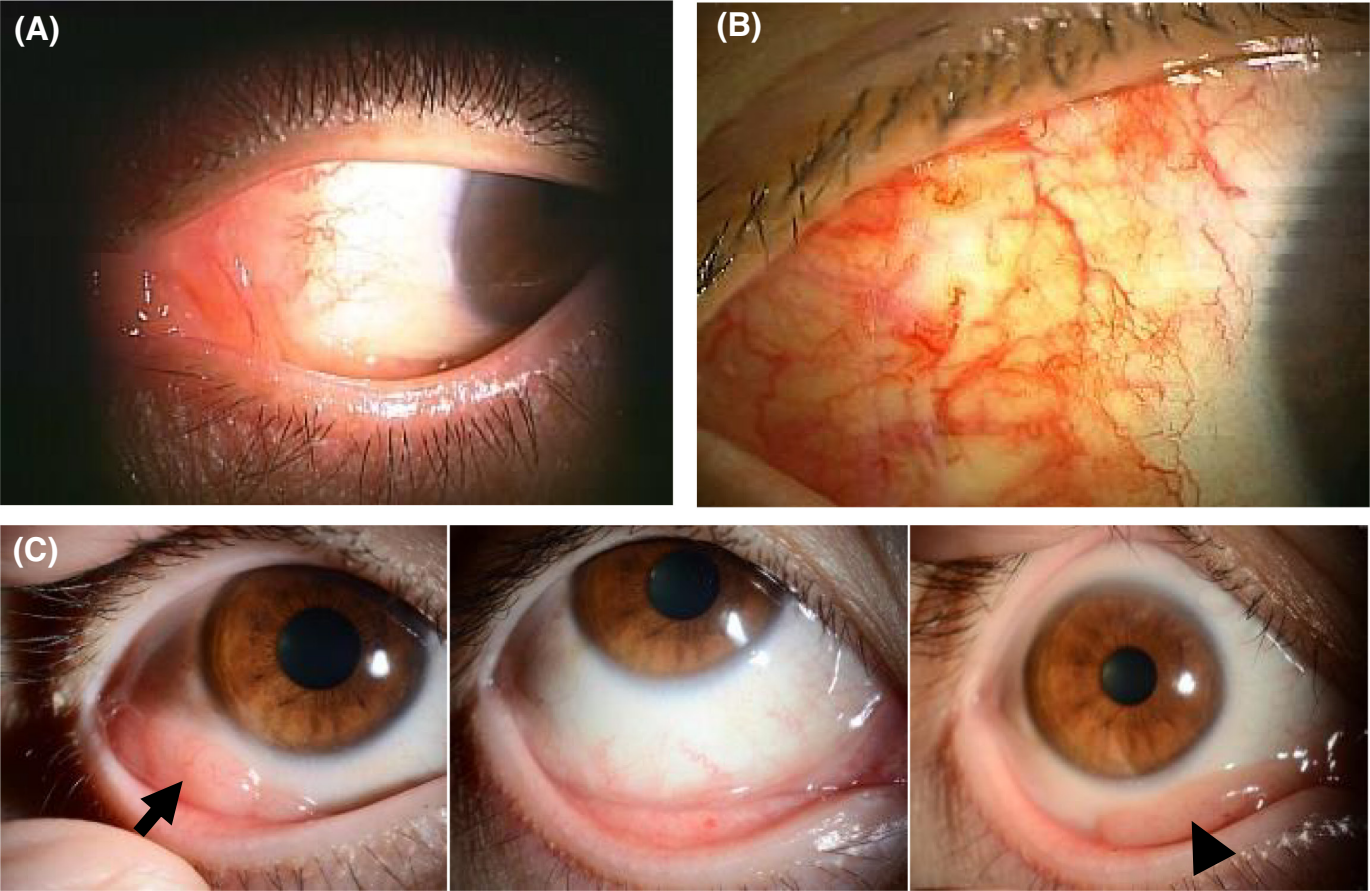
Examples of gross morphologic views of conjunctival lesions. (A) A salmon‐pink patchy lesion involving the bulbar conjunctiva of a 53‐year‐old patient complaining of eye discomfort. The patient was subjected to watchful waiting without progression. (B) A hyperemic lesion of a 63‐year‐old patient who received no treatment without progression. (C) The conjunctival lesion of a 42‐year‐old patient at diagnosis (arrow) (left). The patient had no symptoms and received no treatment after complete resection (middle). The disease relapsed after 3 years of follow‐up (arrow head) (right).

As shown in Table 2, the RT group successfully obtained local disease control. A CR or complete metabolic response (CMR) was achieved in 19 (79.2%) of the 24 patients. The ORR in the R group was 73.7%, and the CR or CMR was 42.1%. However, five patients (26.3%) in the R group failed in achieving an objective response and two of them underwent additional RT. Two patients who received systemic chemotherapy combined with rituximab achieved CR, and one patient with methotrexate withdrawal showed a partial response; these three experienced no disease progression or recurrence during the observation period. Typical morphologic findings of conjunctival lesions in some cases are shown in Figure 1.

**TABLE 2:** Response to initial POAML treatment

Radiotherapy, *n* (%)	(*n* = 24)
CR/CMR	19 (79.2)
PR	5 (20.8)
SD	0
PD	0
ORR	24 (100)
Rituximab monotherapy, *n* (%)	(*n* = 19)
CR/CMR	8 (42.1)
PR	6 (31.6)
SD	5 (26.3)
PD	0
ORR	14 (73.7)
Chemotherapy combined with rituximab, *n* (%)	(*n* = 2)
CR/CMR	2 (100)
PR	0
SD	0
PD	0
ORR	2 (100)
Discontinuation of MTX, *n* (%)	(*n* = 1)
CR/CMR	0
PR	1 (100)
SD	0
PD	0
ORR	1 (100)

Abbreviations: CMR, complete metabolic response; CR, complete response; MTX, methotrexate; ORR, overall response; PD, progressive disease; PR, partial response; POAML, primary ocular adnexal marginal zone lymphoma of mucosa‐associated tissue; SD, stable disease.

### 3.3 Survival outcome

**FIGURE 2:**
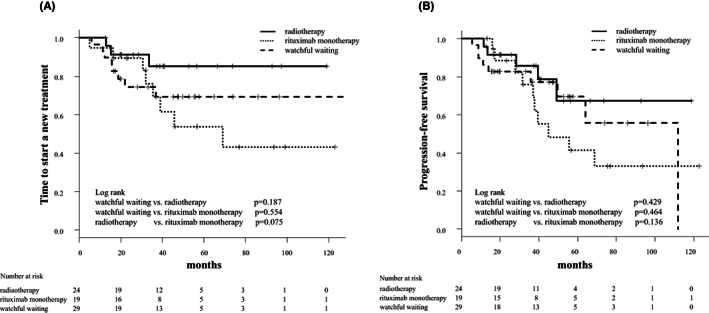
Kaplan–Meier curves for (A) time to start a new treatment (TTNT) and (B) progression‐free survival (PFS) according to treatment modalities. There was no statistically significant difference in both the TTNT and the PFS between treatment modalities.

With a median follow‐up of 48.8 months, the new treatment was added for 20 patients, including 16 with disease progression and four without disease progression, who underwent a new treatment due to their preference. Overall, the median TTNT was not achieved in both WW and RT groups and was 69.0 months in the R group (Figure 2A). The TTNT tended to be longer in the RT group than in the WW and R groups; however, the differences were not statistically significant. At 60 months, the estimated proportion of patients not requiring a new treatment was 69.4% (95% CI: 47.4–83.6) in the WW group, 85.2% (95% CI: 60.2–95.1) in the RT group, and 53.8% (95% CI: 26.0–75.2) in the R group. The median TTNT was 20.3 months (range: 5.9–139.3 months) in the 16 patients with disease progression.

**FIGURE 3:**
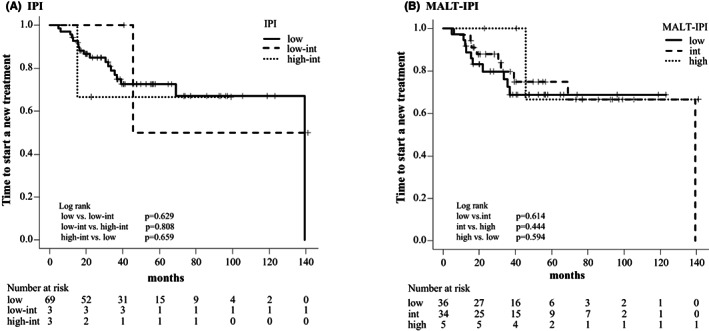
Kaplan–Meier curves of time to start a new treatment (TTNT) according to (A) the IPI and (B) the MALT‐IPI. There was no statistically significant difference in TTNT among different risk groups according to the IPI and the MALT‐IPI score.

The median PFS was not achieved in both WW and RT groups and was 45.0 months in the R group (WW vs. R group, *p* = 0.375; WW vs. RT group, *p* = 0.301; RT vs. R group, *p* = 0.079) (Figure 2B). The 5–year PFS was 69.5% (95% CI: 43.9%–85.2%) in the WW group, 67.4% (95% CI: 34.7%–86.3%) in the RT group, and 41.4% (95% CI: 17.4%–64.1%) in the R group. We found no significant difference in TTNT among different risk groups according to the IPI and the MALT‐IPI (Figure 3). All patients enrolled in this study were alive at the time of analysis, so the OS was 100% during the observation period.

### 3.4 Clinical course and predictive factor for disease progression in the WW group

Of the 29 patients in the WW group, 9 (31.0%) showed disease progression or recurrence, including one patient with systemic progression (Table 3). Of these nine patients, seven received treatments with a median TTNT of 15.6 months (range: 5.9–139.3 months): Three received RT, three underwent surgical resection, and one received rituximab monotherapy, and the disease was well‐controlled in all seven. The remaining two patients continued WW even after disease progression, because of no subjective symptoms or organ impairment. Neither histological transformation nor mortality was observed. In the remaining 20 patients without disease progression, one received RT because of the patient's preference 19 months after diagnosis and one received rituximab monotherapy, expecting an improvement of lacrimation 16 months after diagnosis. At the time of the last follow‐up, 20 (69.0%) of 29 patients did not require treatment.

**TABLE 3:** Characteristics of nine POAML patients who experienced disease progression during WW

Age	Gender	Stage	Laterality	Involved site	Degree of resectionat biopsy	Site of progression	Next treatment
40	F	IE	right	conjunctiva	complete	OA	Surgical resection
42	F	IE	right	conjunctiva	complete	OA	Surgical resection
38	F	IE	left	conjunctiva	complete	OA	Radiotherapy
74	F	IE	left	conjunctiva	complete	OA	Watchful waiting
54	M	IE	left	conjunctiva	partial	OA	Surgical resection
73	M	IE	left	conjunctiva	partial	OA	Radiotherapy
61	M	IE	bilateral	conjunctiva	partial	OA	Watchful waiting
74	M	IE	bilateral	lacrimal gland	partial	OA	Radiotherapy
54	M	IIE	right	lacrimal gland	partial	systemic	Rituximab monotherapy

Abbreviations: F, female; M, male; OA, ocular adnexal region; POAML, primary ocular adnexal marginal zone lymphoma of mucosa‐associated tissue; WW, watchful waiting.

**FIGURE 4:**
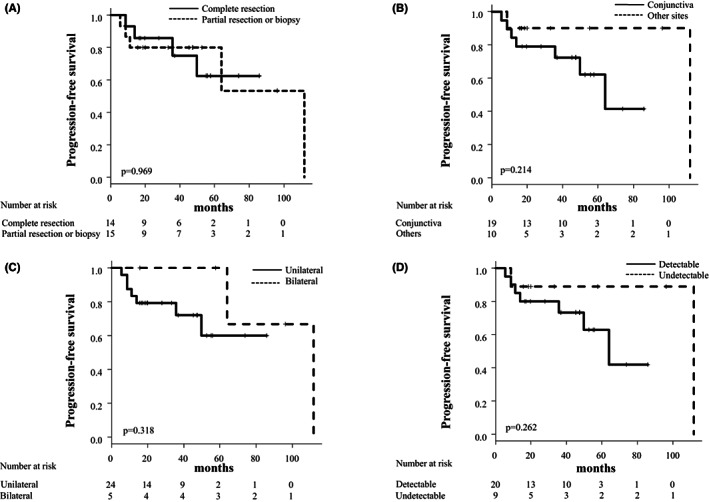
Kaplan–Meier curves of progression‐free survival (PFS) in primary ocular adnexal marginal zone lymphoma of mucosa‐associated tissue (POAML) managed with watchful waiting (WW) according to the degree of resection at biopsy (A), the involved site (B), laterality (C), and the tumor size (D).

To identify predictive factors for disease progression in the WW group, we compared the clinical characteristics of nine patients with disease progression and 20 without disease progression or recurrence. We analyzed age, gender, involved site, laterality, degree of resection, and tumor size evaluated by the detection of post‐biopsy tumor involvement via CT, MRI, or PET‐CT. As shown in Table 4, all these factors were not significantly associated with disease progression or recurrence in the WW group. In addition, the degree of resection on diagnosis, the involved site, laterality, and the tumor size did not significantly affect the PFS in the WW group (Figure 4).

**TABLE 4:** Comparison of characteristics of POAML patients managed with WW with and without disease progression

	No progression (*n* = 20)	Progression (*n* = 9)	*p*
Age			0.185^†^
Median (range)	63 (37–92)	62 (38–74)	
Gender, *n* (%)			0.205*
Male/Female	5 (25)/15 (75)	5 (56) / 4 (44)	
Involved site, *n* (%)			0.431*
Conjunctiva	12 (60)	7 (78)	
Others	8 (40)	2 (22)	
Laterality, *n* (%)			0.633*
Unilateral	17 (85)	7 (78)	
Bilateral	3 (15)	2 (22)	
Degree of resection, *n* (%)			1.000*
Complete resection	10 (50)	4 (44)	
Partial resection or biopsy	10 (50)	5 (56)	
Tumor size, *n* (%)			
Detectable	7 (35)	2 (22)	0.675*
Undetectable	13 (65)	7 (78)	

Abbreviations: IPI, International Prognostic Index; POAML, primary ocular adnexal marginal zone lymphoma of mucosa‐associated tissue; WW, watchful waiting.

* Fisher's exact test.

^†^ Student's *t*‐test.

### 3.5 AEs with RT and rituximab

In the RT group, 16 of 24 patients (66.7%) developed AEs: conjunctivitis (45.8%), radiodermatitis (41.7%), cataract (33.3%), and dry eye (16.7%). Most AEs were generally mild (grade 1 or 2), while grade 3 cataracts occurred in five patients. The median time from RT initiation to onset of cataract was 35 months, and five patients underwent cataract surgery. In the R group, infusion‐related reactions occurred in only 1 of 19 patients (5.3%). No other AEs due to rituximab occurred.

## 4 DISCUSSION

In our study, WW was the most commonly selected therapeutic strategy for POAML patients, and ~70% of patients initially subjected to WW did not experience disease progression or recurrence during 5 years of follow‐up after diagnosis. Previous studies have also supported the clinical utility of WW.^30^, ^31^ Tanimoto et al. demonstrated that ~70% of POAML patients with stage IE disease who were managed with WW did not require any treatment, with a median follow‐up of 7.1 years and survival comparable to that of patients treated by RT immediately after diagnosis.^30^ Another study reported frequent spontaneous regression of POAML, in which patients developed a conjunctiva tumor without any treatment.^31^ These and our results indicate WW as an acceptable approach for POAML treatment, especially in patients without treatment‐emergent subjective symptoms.

The selection of WW as an initial therapeutic approach seemed strongly associated with the absence of treatment‐emergent subjective symptoms as well as the tumor site in a daily practice setting. In the WW group, no patients complained of treatment‐emergent subjective symptoms, such as ptosis, diplopia, or proptosis, and approximately two‐thirds of patients had lymphoma involvement in their conjunctiva. In contrast, 10 (21.7%) of 46 patients in the intervention group had these symptoms. In addition, complete tumor resection at diagnostic biopsy might lead to the selection of WW for initial management. Indeed, 14 (48.3%) of 29 patients in the WW group but only 3 (7.0%) of 46 patients in the RT and R groups had complete tumor resection at biopsy. However, neither the TTNT nor the PFS was significantly affected by the post‐biopsy tumor status, that is, the presence or absence of a residual tumor; a detectable or undetectable tumor by the imaging technique; and the tumor site in the WW group. These results suggest that the clinical outcome by using WW may be comparable to that by administering RT or rituximab monotherapy, even in POAML patients who undergo incomplete tumor resection.

Moreover, as previously reported, no specific clinical information was identified as a predictive factor for the future need for POAML treatment.^30^ Needless to say, tumor debulking, such as by RT, is urgently required when the tumor causes impairment of surrounding tissues and/or visual disturbance. Combined, WW may be a feasible management option, at least in patients with less symptomatic POAML of conjunctival origin.

RT generally provides excellent local disease control in POAML patients.^9^, ^10^, ^11^, ^12^, ^13^, ^14^, ^15^, ^16^ In fact, the ORR by RT was 100% in this study. The main concern with RT in POAML is eye‐related AEs, especially cataracts. The incidence of radiation cataracts requiring surgery has been reported to be 1%–32%,^12^, ^13^, ^14^, ^15^ which is consistent with our result, accounting for 21% of cataract incidence. The favorable utility of lens shielding has been reported for the prevention of RT‐induced cataracts without increasing relapse.^11^, ^12^ Given the excellent visual outcomes following cataract surgery,^32^ RT should not be avoided in POAML patients in case of the need for local control. In contrast, although rituximab monotherapy is considered an alternative treatment option for POAML,^17^, ^18^, ^19^, ^20^ its efficacy remains unclear. In our study, the ORR and CR in the R group were 73.7% and 42.1%, respectively, which were lower than those in the RT group. Furthermore, half of the patients in the R group experienced disease progression and needed new treatment within 4 years. These findings do not support the selection of rituximab monotherapy as the most appropriate treatment strategy for POAML. To achieve excellent local disease control, RT may be the optimal first‐line therapy for symptomatic patients with POAML.

Antibiotic therapy, such as doxycycline, or clarithromycin, is also considered one of the minimally invasive treatment options for POAML patients. However, in Japan, the prevalence of *Chlamydia psittaci* infection in patients with POAML is extremely low and the data on the long‐term efficacy of antibiotics therapy is very limited.^33^ Therefore, antibiotic therapy is not the standard therapeutic strategy in our country.

This study had a few limitations. First, due to the retrospective nature, the non‐uniform treatment plan, and the bias in treatment selection for individual patients, caution is required in interpreting and comparing WW, RT, and rituximab monotherapy treatment outcomes. The WW group had the limited‐stage disease, while the proportion of patients with the bilateral or advanced‐stage disease was higher in the R group. Second, the sample size was small, which potentially decreases statistical power. Third, data on cytogenetic/molecular abnormalities were lacking. Nevertheless, our results support the utility of WW as an initial therapeutic approach for POAML in a real‐world setting and also show the effectiveness of RT, especially if local tumor control is needed.

## 5 CONCLUSION

WW may be an acceptable treatment option for POAML patients, especially asymptomatic patients. RT also plays a primary role in local disease control in POAML. However, further prospective studies with larger sample sizes are required to confirm these results.

## AUTHOR CONTRIBUTIONS

Junya Kuroda, Tsutomu Kobayashi, and Kentaro Mizuhara analyzed and interpreted the data. Junya Kuroda and Tsutomu Kobayashi were involved in the study conception and design. Kentaro Mizuhara, Mitsushige Nakao, Ryoichi Takahashi, Hiroto Kaneko, Kazuho Shimura, Koichi Hirakawa, Nobuhiko Uoshima, Katsuya Wada, Eri Kawata, Reiko Isa, Takahiro Fujino, Taku Tsukamoto, Shinsuke Mizutani, Yuji Shimura, Akiko Yoneda, Akihide Watanabe, and Chie Sotozono were involved in data acquisition. Tsutomu Kobayashi and Kentaro Mizuhara performed the statistical analysis. Kentaro Mizuhara drafted the manuscript. Junya Kuroda and Tsutomu Kobayashi helped with the revision of the manuscript. All authors read and approved the final manuscript.

## CONFLICT OF INTEREST

Junya Kuroda has received research funding from Kyowa Kirin and Chugai Pharmaceutical; has received honoraria from Kyowa Kirin and Chugai Pharmaceutical. T.K. has received honoraria from Chugai Pharmaceutical. Others have no conflict of interest to declare.

## ETHICS APPROVAL

The study was conducted in compliance with the Guidelines for Good Clinical Practice and the Declaration of Helsinki, and the study protocol was approved by the Institutional Review Board of Kyoto Prefectural University of Medicine (protocol code ERB‐C‐424) and institutional review boards of individual institutes. Informed consent was waived because of the retrospective nature of the study and the analysis used anonymous clinical data.

## Data Availability

The datasets used and/or analyzed during the current study are available from the corresponding author on reasonable request.

## References

[1] Mannami T , Yoshino T , Oshima K , et al. Clinical, histopathological, and immunogenetic analysis of ocular adnexal lymphoproliferative disorders: characterization of malt lymphoma and reactive lymphoid hyperplasia. Mod Pathol. 2001;14(7):641‐649. 11454995 10.1038/modpathol.3880366

[2] Cho EY , Han JJ , Ree HJ , et al. Clinicopathologic analysis of ocular adnexal lymphomas: extranodal marginal zone B‐cell lymphoma constitutes the vast majority of ocular lymphomas among Koreans and affects younger patients. Am J Hematol. 2003;73(2):87‐96. 12749009 10.1002/ajh.10332

[3] Olsen TG , Holm F , Mikkelsen LH , et al. Orbital lymphoma‐an international multicenter retrospective study. Am J Ophthalmol. 2019;199:44‐57. 30419193 10.1016/j.ajo.2018.11.002

[4] Hindso TG , Esmaeli B , Holm F , et al. International multicentre retrospective cohort study of ocular adnexal marginal zone B‐cell lymphoma. Br J Ophthalmol. 2020;104(3):357‐362. 31177189 10.1136/bjophthalmol-2019-314008

[5] Nam SW , Woo KI , Kim YD . Characteristics of primary extranodal marginal zone B‐cell lymphoma in Korea: conjunctiva versus other ocular adnexa. Br J Ophthalmol. 2018;102(4):502‐508. 28814410 10.1136/bjophthalmol-2017-310741

[6] Desai A , Joag MG , Lekakis L , et al. Long‐term course of patients with primary ocular adnexal MALT lymphoma: a large single‐institution cohort study. Blood. 2017;129(3):324‐332. 27789481 10.1182/blood-2016-05-714584

[7] Masuda Y , Takeuchi K , Kodama T , et al. Treatment‐associated outcomes of patients with primary ocular adnexal MALT lymphoma after accurate diagnosis. Int J Clin Oncol. 2019;24(12):1620‐1628. 31172332 10.1007/s10147-019-01481-3

[8] Seresirikachorn K , Norasetthada L , Ausayakhun S , et al. Clinical presentation and treatment outcomes of primary ocular adnexal MALT lymphoma in Thailand. Blood Res. 2018;53(4):307‐313. 30588468 10.5045/br.2018.53.4.307 PMC6300677

[9] Galieni P , Polito E , Leccisotti A , et al. Localized orbital lymphoma. Haematologica. 1997;82(4):436‐439. 9299857

[10] Sasai K , Yamabe H , Dodo Y , Kashii S , Nagata Y , Hiraoka M . Non‐Hodgkin's lymphoma of the ocular adnexa. Acta Oncol. 2001;40(4):485‐490. 11504308 10.1080/028418601750288217

[11] Lee J , Oh D , Choi BO , et al. Patterns of care for orbital marginal zone B‐cell lymphoma of mucosa‐associated lymphoid tissue in Korea throughout 2016: results from a multicenter cross‐sectional cohort study (KROG 16‐19). Asia Pac J Clin Oncol. 2019;15(6):358‐363. 31264373 10.1111/ajco.13221

[12] Lee J , Yoon JS , Kim JS , Koom WS , Cho J , Suh CO . Long‐term outcome, relapse patterns, and toxicity after radiotherapy for orbital mucosa‐associated lymphoid tissue lymphoma: implications for radiotherapy optimization. Jpn J Clin Oncol. 2019;49(7):664‐670. 31505651 10.1093/jjco/hyz044

[13] Lee GI , Oh D , Kim WS , et al. Low‐dose radiation therapy for primary conjunctival marginal zone B‐cell lymphoma. Cancer Res Treat. 2018;50(2):575‐581. 28618774 10.4143/crt.2017.182 PMC5912130

[14] Uno T , Isobe K , Shikama N , et al. Radiotherapy for extranodal, marginal zone, B‐cell lymphoma of mucosa‐associated lymphoid tissue originating in the ocular adnexa: a multiinstitutional, retrospective review of 50 patients. Cancer. 2003;98(4):865‐871. 12910532 10.1002/cncr.11539

[15] Ejima Y , Sasaki R , Okamoto Y , et al. Ocular adnexal mucosa‐associated lymphoid tissue lymphoma treated with radiotherapy. Radiother Oncol. 2006;78(1):6‐9. 16359744 10.1016/j.radonc.2005.11.005

[16] Kim SE , Yang HJ , Yang SW . Effects of radiation therapy on the meibomian glands and dry eye in patients with ocular adnexal mucosa‐associated lymphoid tissue lymphoma. BMC Ophthalmol. 2020;20(1):24. 31931766 10.1186/s12886-019-1301-0 PMC6958586

[17] Ferreri AJ , Ponzoni M , Martinelli G , et al. Rituximab in patients with mucosal‐associated lymphoid tissue‐type lymphoma of the ocular adnexa. Haematologica. 2005;90(11):1578‐1579. 16266908

[18] Mino T , Mihara K , Yoshida T , Takihara Y , Ichinohe T . Monthly administration of rituximab is useful for patients with ocular adnexal mucosa‐associated lymphoid tissue lymphoma. Blood Cancer J. 2014;4(9):e245. 25215661 10.1038/bcj.2014.65 PMC4183774

[19] Fujita Y , Mimura M , Satou Y , Akioka T , Oku H , Ikeda T . Rituximab monotherapy for compressive optic neuropathy with giant ocular adnexal mucosa‐associated lymphoid tissue lymphoma. Ophthal Plast Reconstr Surg. 2021;37(3S):S132‐S133. 10.1097/IOP.0000000000001803 32826825

[20] Tuncer S , Tanyıldız B , Basaran M , Buyukbabani N , Dogan O . Systemic rituximab immunotherapy in the Management of Primary Ocular Adnexal Lymphoma: single institution experience. Curr Eye Res. 2015;40(8):780‐785. 25247376 10.3109/02713683.2014.959605

[21] Kim SY , Yang SW , Lee WS , et al. Frontline treatment with chemoimmunotherapy for limited‐stage ocular adnexal MALT lymphoma with adverse factors: a phase II study. Oncotarget. 2017;8(40):68583‐68590. 28978139 10.18632/oncotarget.19788 PMC5620279

[22] Ferreri AJ , Sassone M , Miserocchi E , et al. Treatment of MALT lymphoma of the conjunctiva with intralesional rituximab supplemented with autologous serum. Blood Adv. 2020;4(6):1013‐1019. 32182364 10.1182/bloodadvances.2020001459 PMC7094013

[23] Ferreri AJ , Ponzoni M , Guidoboni M , et al. Regression of ocular adnexal lymphoma after chlamydia psittaci‐eradicating antibiotic therapy. J Clin Oncol. 2005;23(22):5067‐5073. 15968003 10.1200/JCO.2005.07.083

[24] Nastoupil LJ , Sinha R , Byrtek M , et al. Outcomes following watchful waiting for stage II‐IV follicular lymphoma patients in the modern era. Br J Haematol. 2016;172(5):724‐734. 26729445 10.1111/bjh.13895

[25] Ardeshna KM , Qian W , Smith P , et al. Rituximab versus a watch‐and‐wait approach in patients with advanced‐stage, asymptomatic, non‐bulky follicular lymphoma: an open‐label randomised phase 3 trial. Lancet Oncol. 2014;15(4):424‐435. 24602760 10.1016/S1470-2045(14)70027-0

[26] Ardeshna KM , Smith P , Norton A , et al. Long‐term effect of a watch and wait policy versus immediate systemic treatment for asymptomatic advanced‐stage non‐Hodgkin lymphoma: a randomised controlled trial. Lancet. 2003;362(9383):516‐522. 12932382 10.1016/s0140-6736(03)14110-4

[27] Thieblemont C , Cascione L , Conconi A , et al. A MALT lymphoma prognostic index. Blood. 2017;130(20):1409‐1417. 28720586 10.1182/blood-2017-03-771915

[28] Cheson BD , Fisher RI , Barrington SF , et al. Recommendations for initial evaluation, staging, and response assessment of Hodgkin and non‐Hodgkin lymphoma: the Lugano classification. J Clin Oncol. 2014;32(27):3059‐3068. 25113753 10.1200/JCO.2013.54.8800 PMC4979083

[29] Kanda Y . Investigation of the freely available easy‐to‐use software ‘EZR’ for medical statistics. Bone Marrow Transplant. 2013;48(3):452‐458. 23208313 10.1038/bmt.2012.244 PMC3590441

[30] Tanimoto K , Kaneko A , Suzuki S , et al. Long‐term follow‐up results of no initial therapy for ocular adnexal MALT lymphoma. Ann Oncol. 2006;17(1):135‐140. 16236754 10.1093/annonc/mdj025

[31] Matsuo T , Yoshino T . Long‐term follow‐up results of observation or radiation for conjunctival malignant lymphoma. Ophthalmology. 2004;111(6):1233‐1237. 15177977 10.1016/j.ophtha.2003.09.049

[32] Clark A , Morlet N , Ng JQ , Preen DB , Semmens JB . Whole population trends in complications of cataract surgery over 22 years in Western Australia. Ophthalmology. 2011;118(6):1055‐1061. 21310493 10.1016/j.ophtha.2010.11.001

[33] Travaglino A , Pace M , Varricchio S , et al. Prevalence of chlamydia psittaci, chlamydia pneumoniae, and chlamydia trachomatis determined by molecular testing in ocular adnexa lymphoma specimens. Am J Clin Pathol. 2020;153(4):427‐434. 31755895 10.1093/ajcp/aqz181

